# Pepsinogen C expression, regulation and its relationship with cancer

**DOI:** 10.1186/s12935-017-0426-6

**Published:** 2017-05-23

**Authors:** Shixuan Shen, Jingyi Jiang, Yuan Yuan

**Affiliations:** Tumor Etiology and Screening Department of Cancer Institute and General Surgery, The First Affiliated Hospital of China Medical University, Key Laboratory of Cancer Etiology and Prevention of Liaoning Provincial Education Department, Shenyang, 110001 China

**Keywords:** Pepsinogen C, Gastric cancer, Expression, Regulation, Sex hormone

## Abstract

Pepsinogen C (PGC) belongs to the aspartic protease family and is secreted by gastric chief cells. PGC could be activated to pepsin C and digests polypeptides and amino acids, but as a zymogen PGC’s functions is unclear. In normal physiological conditions, PGC is initially detected in the late embryonic stage and is mainly expressed in gastric mucosa. The in situ expression of PGC in gastric mucosa is decreased considerably in the process of superficial gastritis → atrophic gastritis → gastric cancer (GC), proving that PGC is a comparatively ideal negative marker of GC. Serum PGC, and PGA levels and the PGA/PGC ratio have satisfactory sensitivity, specificity and price–quality ratio for predicting high GC risk. Ectopic PGC expression is significantly increased in prostate cancer, breast cancer, ovary cancer and endometrial cancer. In those sex-related cancers high level PGC expression indicates better prognosis and longer survival. The regulation of PGC expression involves genetic and epigenetic alteration of the encoding *PGC* gene, hormones modulation and interactions between PGC with other transcription factors and protein kinases. More and more research evidence hinted that PGC has strong correlation with cancer. In the systematic review, we respectively elaborate the structure, potential physiological functions, expression characteristics and regulation of PGC, and especially focus on the relationship between PGC expression and cancer to highlight the role of PGC in the tumorigenesis and its application value in clinical practice.

## Background

Pepsinogen (PG) is the precursor of pepsin and is composed of five hypotypes: pepsinogen A (PGA), pepsinogen B (PGB), pepsinogen C (PGC), pepsinogen F (PGF) and chymosinogen. PGA is expressed in the gastric body throughout a human’s lifespan. PGB is found only in pigs. Chymosinogen and PGF are expressed in fetuses and infants. PGC appears in the late stage of embryonic development and its expression is more extensive, which destines PGC as a mature sign of organ differentiation. Compared with the other PGs, PGC has unique physiological and pathological characteristics.

PGC belongs to the aspartic protease family, and is synthesised by chief cells of the gastric mucosa and secreted to the stomach cavity. Under acid conditions, PGC is activated to pepsin C. Activated PGC digests polypeptides and amino acids, but whether it functions as a zymogen is unclear. In normal physiological conditions, PGC is mainly expressed in the entire stomach as a final product of mature differentiated gastric mucosa cells [1]. About 1% of PGC enters the blood circulation in stable form through capillaries in the gastric mucosa. Ectopic PGC expression has been confirmed in the prostate, lungs, seminal vesicles and seminal fluid [2–4]. In pathological conditions, PGC expression levels change significantly. The in situ expression of PGC in the gastric mucosa is decreased considerably or even absent in the dynamic process of superficial gastritis (SG) → atrophic gastritis (AG) → gastric cancer (GC), showing that PGC is a comparatively ideal negative marker of GC. Serum PGC and its combination with PGA and the PGA/PGC ratio have a satisfactory sensitivity, specificity and price–quality ratio for predicting individuals at high risk for GC. Ectopic PGC expression levels are significantly increased in prostate cancer, breast cancer and ovary cancer [5–8]. In those sex-related cancers, high level PGC expression indicates a better prognosis and longer survival. The regulation of PGC expression involves genetic and epigenetic alteration of the encoding *PGC* gene, hormones control and interactions among PGC with other transcription factors and protein kinases. Recently, more and more research evidences have been found and hint that PGC has strong correlation with cancer. In the present review, we systematically describe the structure, potential physiological functions, expression characteristics and its regulation of PGC, and especially focus on the relationship between PGC expression and cancer to highlight the role of PGC in the tumorigenesis and its application value in clinical practice.

## The structure and function of PGC

**Fig. 1:**
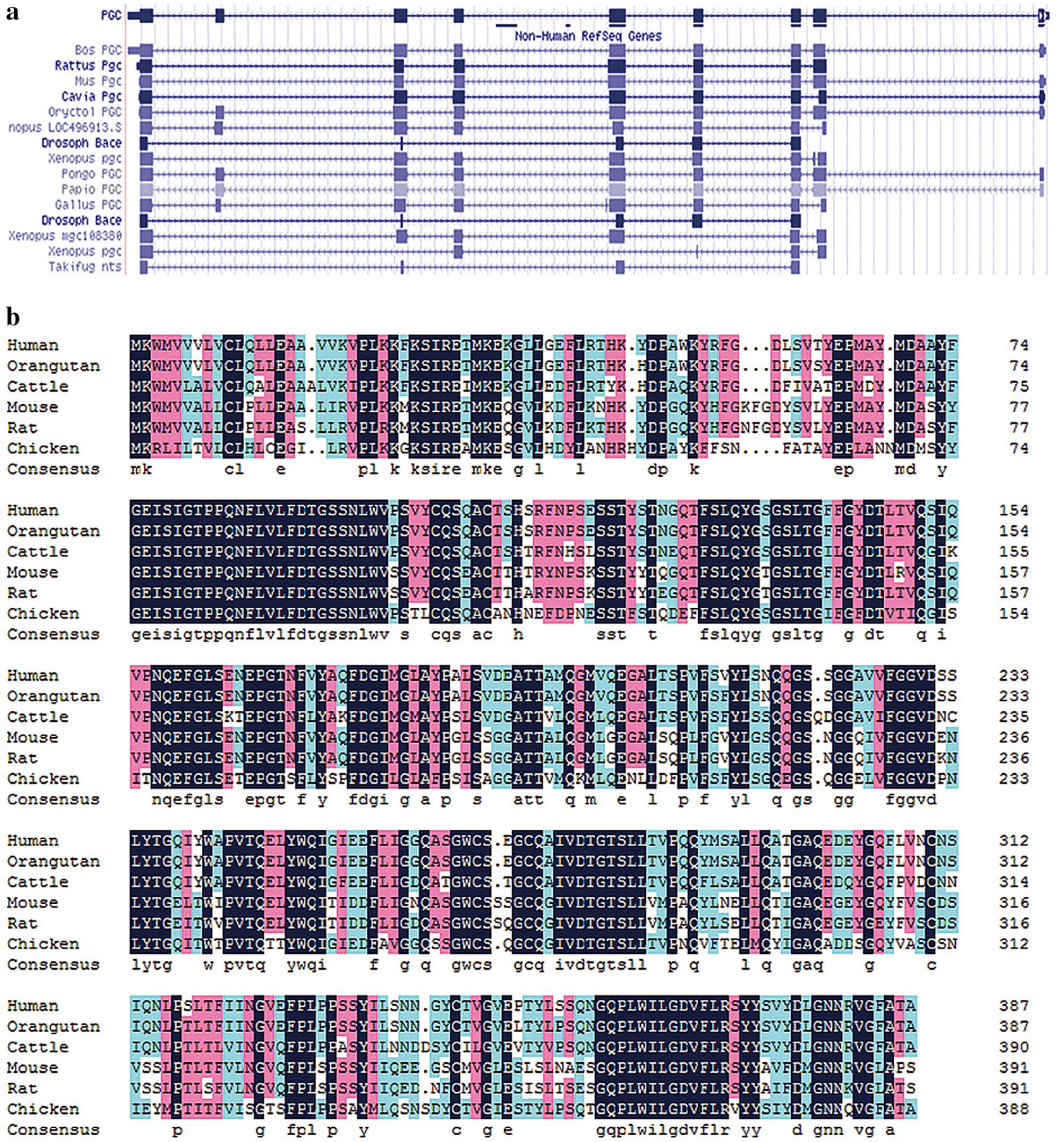
The conserved sequence of PGC gene among various species. The areas of *dark blue* are the same sequences. **a** The sequence of PGC mRNA sequence. **b** The sequence of PGC protein

The *PGC* gene is located at chromosome 6p21.1 with 11 exons, encoding a 42 kDa PGC protein. It is present in various species, including humans, pigs, rats, rabbits, chicken and fish. Across different species, it has a highly conserved sequence evolved from a common pepsin-like ancestral gene with about 80% sequence similarity [9] (Fig. 1). According to NCBI (National Center for Biotechnology Information), Ensembl, Vega data and Uniprot, there are four splice variants of *PGC* mRNA. Only transcript 1 and transcript 2 could encode PGC protein named isoform 1 and isoform 2. Isoform 1 (molecular weight 42 kDa) contains the overall length of the *PGC* gene encoding information, and it is known as the most common subtype of PGC protein; isoform 2 is a polypeptide chain with 315 amino acids and a molecular weight of 34 kDa. Other isoforms have not yet been confirmed on the basis of bioinformatic prediction. The primary structure of PGC protein is a polypeptide chain with 388 amino acid residues. Among them, 329 amino acids comprise the mature pepsin C. PGC amino acid sequence is divided into three regions: propeptide (43 amino acid residues), activation fragments (16 amino acid residues) and activating enzyme (329 amino acid residues) [10]. The PGC secondary structure is 15% α-helix and 44% β-sheet [1], illustrating PGC is β-sheet protein–rich. The PGC protein structure achieves stability through numerous hydrogen bonds; in addition, its *N*-glycosylation sites also minimise protein conformational alteration to stabilise the protein structure [11]. X-ray crystallography data had shown that the PGC protein tertiary structure was similar to the aspartic protease family and that it contained a plurality of aspartic protease family symbols and two rhodopsin-like G protein–coupled receptor symbols. The PGC tertiary structure is the symmetrical double-lobe type and the centres of the two lobes contain two aspartate activated substrate binding sites (Asp32 and Asp215), which can accommodate seven–amino acid residue substrates. The PGC S1 sub-binding sites are the main determinant of substrate specificity. Their basic characteristic is the presence of a Leu71–Gly82 ring around Asp32, which is comprised of some hydrophobic groups.

**Fig. 2:**
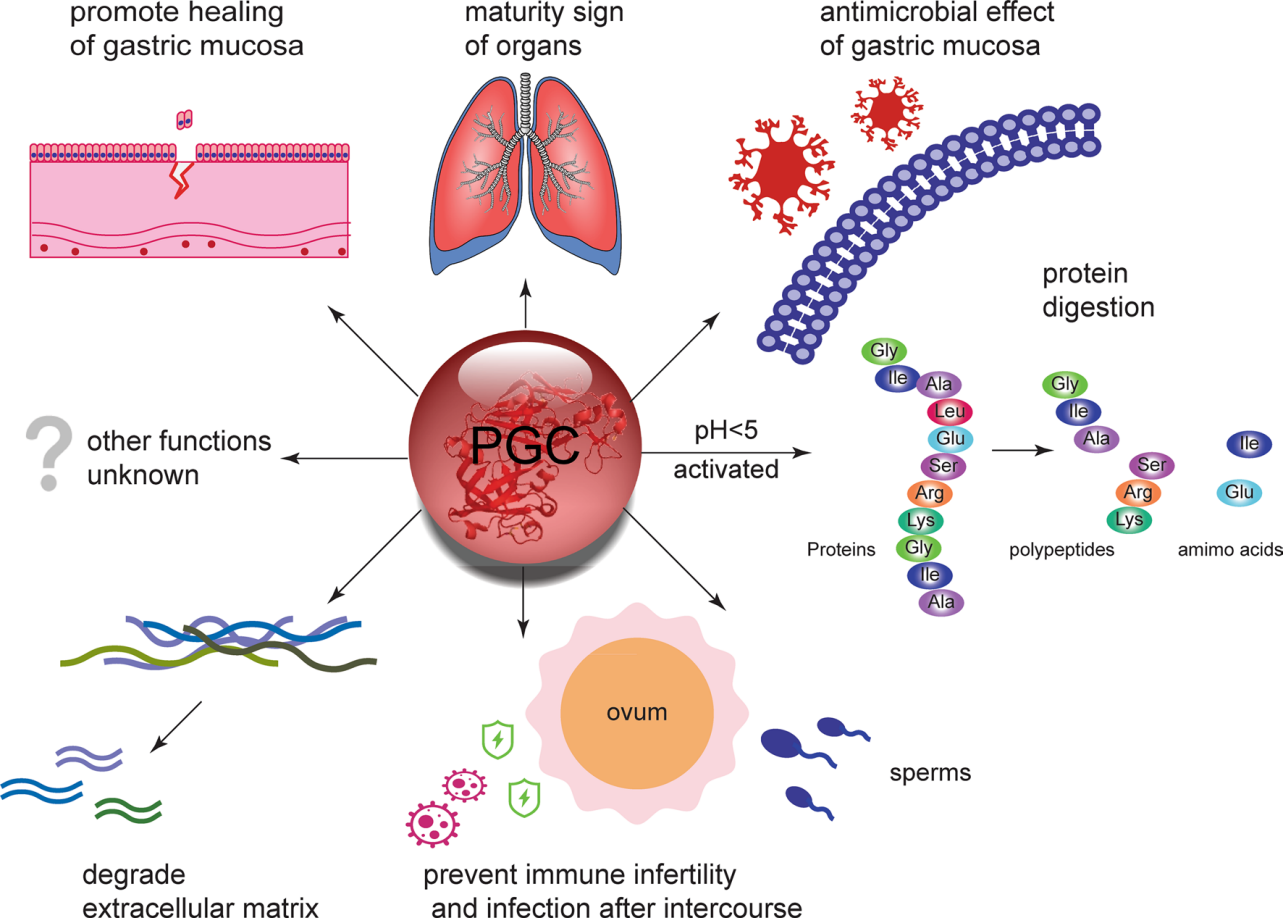
Potential physiological functions of PGC. The functions of PGC includes digesting proteins, promoting the healing of gastric mucosa, maturing of organs, antimicrobial effect of gastric mucosa, preventing immune infertility and infection after intercourse, and degrading extracellular matrix. Many other functions of PGC are still unknown

PGC is an endoprotease of the aspartic protease family and is secreted by chief cells. During the resting state, PGC is stored in zymogen granules and its synthesis was halted, whereas it is secreted to the stomach cavity under stimulation by physiological or external chemical signals. PGC is stable in neutral pH conditions; at pH < 5, it becomes an active enzyme. Zymogen activation is a complicated process that involves many conformational changes and chemical bond fractures, eventually resulting in exposure of the active site and excision of the propeptide. As an active enzyme, pepsin C functions in digesting proteins to polypeptides and amino acids, whereas the features of PGC as a zymogen have not been clarified. Through immunohistochemistry, Elabiad et al. detected PGC expression only in mature fetuses but not in fetuses younger than 23 weeks gestational age [12]. This indicated that PGC appeared in late embryogenesis, which is a critical sign of maturity in well-differentiated organs. Feng et al. found that, in orange-spotted grouper, PGC was first expressed at 41 days after hatching and continuously expressed until adulthood in the stomach mucosa, illustrating the fact that PGC affects the development [13]. Fukuda et al. elucidated that embryonic chicken pepsinogen gene transcripts were detected only in embryonic proventriculus epithelial cells of glandular structures. However, its expression was completely ceased in the proventriculus of chick 2 weeks after hatching. The results demonstrated that expression of the pepsinogen gene was strictly cell-type-specific and developmental stage-specific at the level of transcription [14]. In a study of a fragment of a synthetic of rat PGC, Kishi et al. found that PGC disrupted the growth of normal RGM-1 rat cells and PGC expression was enhanced in acetic acid–induced ulcers, indomethacin-induced gastric lesions and *Helicobacter pylori* infection status. This suggested that PGC played a role in healing of the gastric mucosa [15]. Minn et al. isolated three polypeptides with broad-spectrum antibiotic activity from bullfrog stomach and found that two of them were from the N-terminal of PGA or PGC. Synthetic human or monkey PGC had similar activity, which indicated that these polypeptides might have an antimicrobial effect on the gastrointestinal mucosa [16]. Gerson et al. verified that PGC in the acidic organelles hydrolyses pro-surfactant protein B (pro-SPB), which is secreted by alveolar type 2 epithelial cells, and matures it through zymogen activation. If the *PGC* gene was knocked down, pro-SPB maturation would be inhibited [17]. PGC is also a valuable marker for distinguishing transdifferentiated type 1 epithelial cells [3]. These evidence indicate that PGC also plays a major role in lung maturation. Sorensen et al. reported that hCAP (humancathelicidin) was a major protein of the specific granules of human neutrophilsin seminal fluid, which produces antimicrobial peptides under the influence of PGC after sexual intercourse. The enzyme activation following exposure of the antimicrobial peptides in seminal fluid to the vaginal fluids indicated that PGC was involved in the mechanism of infection prevention after sexual intercourse [18]. In addition, PGC might also function in reproduction. Szecsi et al. verified that the acidic conditions in the vagina activated PGC and degraded other sperm proteins to reduce the vaginal immune load. These processes contribute to the prevention of immune infertility [19]. Dos Reis et al. have revealed that PGC degraded the extracellular matrix. Proteolytic enzymes are associated with tumour invasion and metastasis. Similar to other proteases such as matrix metalloproteinase and plasminogen activator, PGC might exert its dissolving function in tumour cells [20]. The schema chart of potential physiological functions of PGC was drawn in Fig. 2.

## PGC expression and cancer

Human PGC is expressed in at least three forms, including gastric mucosal in situ, serum and ectopic expression. Under pathological conditions, PGC expression levels change significantly and correlate closely with cancer development and progression.

### In situ PGC expression and GC

PGC in situ expression occurs mainly in the normal gastric mucosa and the expression is decreased or absent during GC development. Ning et al. detected PGC expression in different gastric diseases by immunohistochemistry and discovered positive PGC expression in normal gastric mucosa (100%), whereas its positivity declined considerably (2.4%) in GC. In the process of SG or gastric erosive ulcer → AG or dysplasia → GC, the rate of positive PGC expression declines gradually [21, 22]. These findings suggested that PGC in situ expression levels correlated negatively with GC occurrence. Melle et al. screened differentially expressed proteins between normal gastric mucosa and GC tissue using the proteomics approach, subsequently confirmed with 2D electrophoresis and immunohistochemistry, and they found that there were greatest differences of PGC expression in the both [23]. Xu et al. detected PGC, mucin 1 (MUC1) and MUC2 co-expression in SG → AG → GC process and found that PGC staining was positive in SG and AG while it was negative in GC. The PGC(−) MUC1(−) MUC2(+) phenotype could distinguish mucinous adenocarcinoma or signet ring cell carcinoma from ductal adenocarcinoma with mucin secretion [24]. Tatematsu et al. observed that PGC was expressed in chief cells, mucous neck cells of the fundic glands, and also in pyloric gland cells and Brunner’s gland cells [25]. Steele et al. also used PGC to represent the normal status of gastric mucosa cells in human and mouse [26]. Thus, PGC could serve as a symbol of primary gastric epithelial cells. In addition, Stemmermann et al. observed PGC expression in various degrees of intestinal metaplasia and GC. A possible reason for PGC positive expression in intestinal metaplasia–related tumours may be that the residual normal stomach glandular bodies or intestinal metaplasia cells induced a gastric phenotype mutation [27]. Fernadez et al. detected PGC expression in 95 GC cases and followed up for 21.4 months to assess the relation of PGC expression level with prognosis. They found 26.3% positive PGC staining in GC, and the proportion of PGC-positive GC cells in well-differentiated tumours (50%) was significantly higher than that in moderately differentiated tumours (19.5%) and poorly differentiated tumours (21.9%). And low PGC expression was associated with shorter overall survival of GC. Thus, they concluded that PGC expression is an independent prognostic factor [28].

### Serological PGC expression and cancer

#### Serological PGC expression and mucosal inflammation or *H. pylori* infection

Generally, about 1% of PGC enters the blood circulation through the gastric mucosa capillaries in a stable form. Once *H. pylori* infects or damages the mucosa, the serum PGC concentration changes, which reflects the status of *H. pylori* infection and mucosal inflammation. He et al. demonstrated that serum PGC concentrations were quite low in the healthy population [29]: The average level was 6.6 mg/L, and was higher in men than in women (men, 7 mg/L; women, 6 mg/L). The cut-off values of serum PGC varied by geographic region and ethnicity [30]. In patients with SG or SG with intestinal metaplasia, the serum PGC levels of serologically *H. pylori*–positive patients were higher than that of *H. pylori*–negative patients [29]. Serum PGC could serve as an effective biomarker for monitoring changes in gastric morphology before and after the eradication of *H. pylori*. Massarrat et al. compared serum PGA and PGC levels before and 2.5 years after *H. pylori* eradication. They concluded that serum PGA and PGC declined to 70 and 45% of the previous levels after *H. pylori* had been eliminated, respectively, and patients with mononuclear cell infiltration had lower PGA levels and higher PGC levels [31]. Using enzyme-linked immunosorbent assay, Sun et al. observed remarkably reduced serum PGA and PGC levels and significantly increased PGA/PGC ratio after bacterial eradication therapy in patients with *H. pylori*-related disease. Therefore, serum PGC detection is suitable for determining the eradication efficacy of *H. pylori*, and the PGA/PGC ratio can act as an assessment criterion of eradication efficacy in the early stage of infection [32]. Kiyohira et al. considered serum PGC ≥ 12 ng/mL or PGA/PGC ratio ≤4.0 as the cut-off values for diagnosing *H. pylori* infection, with 90.0% sensitivity and 93.5% specificity. Patients with serum PGA ≥ 85 ng/mL or serum PGC ≥ 15 ng/mL were *H. pylori*–positive; patients with PGA/PGC ratio > 6.5 were *H. pylori*–negative [33].

#### Serological PGC expression and GC

Several studies have demonstrated that serum PGC combined with serum PGA and the PGA/PGC ratio can be used not only for assessing *H. pylori* infection but also for screening individuals at high risk of AG and GC. Serum PGC serves as a biomarker of gastric mucosa morphology and precancerous lesions. Samloff et al. reported that serum PGA and PGC levels and the PGA/PGC ratio could be used as serological biopsy guides to reflect the morphology and function of gastric mucosa [34]. Serum PGC concentration increased gradually with the exacerbation of gastric disease from SG → SG with intestinal metaplasia → AG → dysplasia → GC [35]. Miki et al. used serum PGA < 50 mg/L and PGA/PGC ratio <3.0 in a comprehensive screen of GC in Japan [36]. Sun et al. reported that a PGA/PGC ratio <7 is suitable for identifying populations at high risk of GC in Chinese [37]. Tu et al. evaluated temporal changes in PGA and PGC levels and PGA/PGC ratio with the risk for progression of gastric precancerous lesions and suggested that increased serum PGA and PGC and decreased PGA/PGC ratio were associated with risk for progression of gastric precancerous lesions [38]. Xie et al. found that the linear range for detecting PGC was 2.5–80 ng/mL by electrochemical microfluidic chip method. This method for PGC detection has great application value for early screening of GC patients, therapeutic evaluation, and real-time dynamic monitoring of GC progression. The multi-index prediction model can be used to predict risk of GC more accurately and effectively. Through this method we could further confirmed the diagnosis value of PGC in GC [39]. Cao et al. found that PGC was a preferable diagnostic biomarker of GC. Patients with GC or gastric atrophy had higher PGC levels and lower PGA/PGC ratio as compared to healthy controls [40]. Huang et al. conducted a meta-analysis to assess the value of serum PGC as a biomarker for screening AG and GC. They suggested that the sensitivity and specificity of combined detection of PGA level and PGA/PGC ratio for screening GC was 0.70 [95% confidence interval (CI) 0.66–0.75] and 0.79 (95% CI 0.79–0.80), respectively. The sensitivity and specificity for screening AG was 0.79 (95% CI 0.72–0.85) and 0.89 (95% CI 0.85–0.93), respectively [41]. More recently, Yeh et al. assessed the screening and treatment efficiency of serum PGA and PGC, endoscopy and *H. pylori* infection through a microsimulation model of American intestinal-type non-cardia gastric adenocarcinoma (NCGA). The results indicated that the risk of intestinal-type NCGA could be reduced by screening the general population at 50 years of age as follows: serum PG screening, 26.4%; endoscopic screening and endoscopic submucosal dissection, 21.2%; *H. pylori* screening and treatment, 0.2%. Consequently, serum PG screening was more effective than the other methods. It was an efficient measure for decreasing the mortality rate of smokers with intestinal metaplasia–type NCGA in the high-risk GC group. Accordingly, a more ideal sequential screening strategy should start with serum PG detection, followed by endoscopic biopsy to screen the positive results. This could contribute to distinguish high-risk individuals from low-risk individuals to ensure that they will benefit from subsequent therapy [42].

#### Serological PGC expression and other cancers

Serum PGC is related with cancers of other organs beyond initial gastric diseases. Ito et al. first discovered the correlation between pancreatic intraductal papillary mucinous tumours (IPMN) and serum PGC. Patients with gastric-type IPMN had higher serum PGC levels, which decreased after pancreaticoduodenectomy [43]. Yada et al. reported that duodenal tumours enhanced serum PGC abnormally (168.8 ng/mL), which decreased after tumour resection. As the patients did not have any obvious gastric inflammation, atrophic changes or *H. pylori* infection, their serum PGC should be in the normal range. The preoperative aberrant elevated serum PGC may be due to obstruction of the duodenal Brunner glands by the large duodenal tumour, and PGC was more likely to be secreted to the circulation [44]. Esophageal squamous dysplasia (ESD) is a precancerous lesion of esophageal squamous cell carcinoma (ESCC). The serum PGA/PGC ratio is significantly lower in individuals with ESD than in those without such precancerous lesions. PGA/PGC levels correlated negatively with ESD risk and incidence and correlated positively with the gastrointestinal bacterial flora numbers [45, 46].

### Ectopic PGC expression and cancer

Although PGC expression in vivo is mainly in the stomach, there are several reports on ectopic PGC expression in extragastric organs such as the prostate, lungs and seminal vesicles [2–4]. PGC expression levels were apparently promoted, especially in the tumour, for example, in prostate cancer, breast cancer, ovary cancer, endometrial cancer, pancreatic cancer, kidney cancer, bladder cancer, eyelid basal cell carcinoma, squamous cell carcinoma and melanoma, etc. [5, 7, 8, 47–50]. It is worth noting that in sex-related tumours such as prostate cancer, breast cancer and ovary cancer, PGC expression is increased in cancer tissues as compared to normal tissues. Using immunohistochemistry, Diaz et al. concluded that 42.8% of patients with stage D2 prostate cancer were PGC-positive and that PGC was a valuable prognostic factor that indicated better prognosis and longer survival [2]. Antunes et al. reported that PGC was overexpressed in 72.7% of prostate cancer cases, and its increased expression was often accompanied by the aggravation of clinical symptoms [5]. Serra et al. using immunohistochemical staining showed that about 46% of breast cancers were PGC-positive, and the degree of PGC expression in men with breast cancer was higher than that in women with breast cancer. As an aspartic protease, PGC might contribute to the decomposition of invasive breast cancer lesions [6, 7]. The consensus is that PGC expression in breast cancer cells might represent good prognosis. A possible explanation for this is that the presence of PGC may affect hormone receptor pathways. Balbin et al. reported that PGC was also expressed in breast cancer. The observed extragastric expression of PGC may be a consequence of the ability of this gene to respond to the hormonal stimuli, including androgens, glucocorticoids, and progesterone [51]. Rojo et al. proved that, in patients with ovary cancer with serum CA125 < 35 U/mL, PGC was a good prognostic factor expressed in about 25% of patients with ovary cancer [8]. Tenti et al. studied nine biomarkers of gastric, intestinal and pancreatic ductal epithelium cells in mucinous ovarian adenocarcinoma and found that PGC expression levels in ovary benign tumours and borderline tumours were higher than that in ovary cancer (*P* < 0.005) [52]. Rothacker et al. also reported PGC expression in ovarian mucinous cystadenoma to prove gastric epithelial differentiation [53]. Fukushima et al. compared the differential gene expression between the normal pancreatic ductal epithelial cells and mucinous cystic neoplasms cells, and found that *PGC* was one of the 114 genes overexpressed in neoplasms cells through oligonucleotide microarray analysis [54]. Miyasaka et al. found that PGC is overexpressed in hepatic carcinoma cells (HCC) as well. Although the role of PGC in HCC is still unknown, the common pathway of tumour growth or tumorigenesis between HCC and other PGC-producing cancers may be found [55]. Nakamura et al. proved that PGC expression in tumour cells represented their normal secretion function. And downregulating PGC caused tumour cell dedifferentiation or deterioration, which is closely related with cancer prognosis and metastasis [56].

## The regulation of PGC expression

**Fig. 3:**
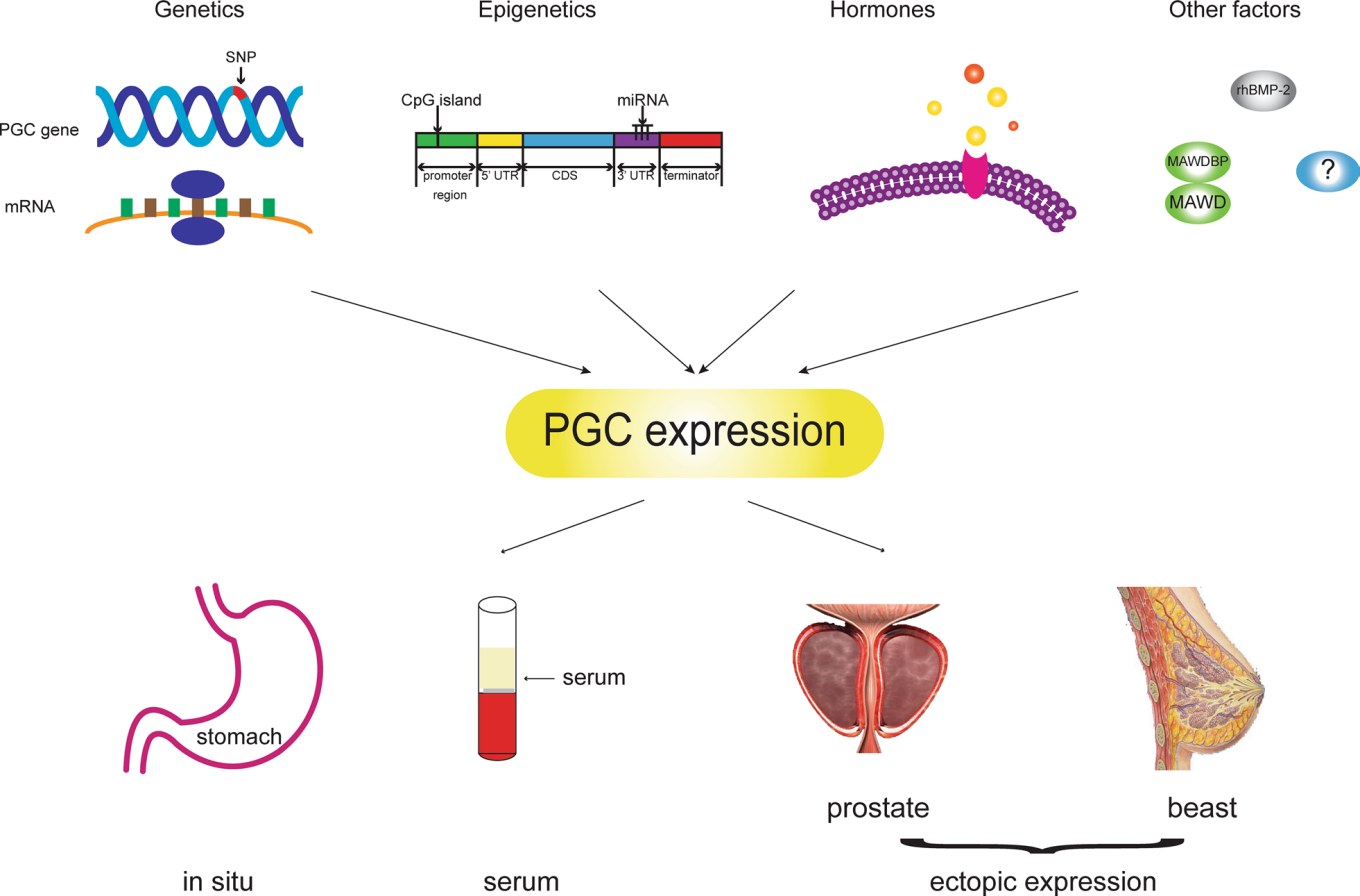
The regulation of PGC expression. The regulation of PGC expression involves genetic and epigenetic alteration of the encoding gene, hormones modulation and interactions between PGC with other transcription factors and protein kinases

The regulation of gene expression refers to a regulative progress of a gene expressed from DNA to protein . And the regulation of PGC expression involves genetic and epigenetic alteration of the encoding gene, hormones control and interactions between PGC with other transcription factors and protein kinases (Fig. 3).

### The regulation of PGC expression via the PGC gene

#### Genetic alteration of PGC gene affects PGC expression

Human *PGC* is a 10.7-kbp single-copy gene located on 6p21.1. with 11 exons. The gene has several forms of genetic variation, including insertion/deletion polymorphism and single-nucleotide polymorphisms (SNPs), which affect PGC expression and regulation. In 1988, Azuma et al. showed that there was a 100-bp insertion/deletion polymorphism between exon 7 and 8 of the *PGC* gene. Based on the fragment lengths, four alleles with different molecular weights were obtained following agarose gel electrophoresis of PCR products: allele 1 (310 bps) allele 2 (400 bps), allele 3 (450 bps) and allele 4 (480 bps) [57]. Liu et al. reported that PGC *Eco*RI length polymorphism was associated with risk of peptic ulcer and GC that homozygous allele 1 downregulated PGC expression. Liu and Sun detected the PGC genotype in patients with or without GC, and found that PGC homozygous allele 1 and PGC expression were negatively correlated [58, 59]. From homozygous allele 1 → heterozygous allele 1 → other types, the rate of positive PGC expression increased gradually. In recent years, Kumarh as used similar methods and reached further conclusions that PGC homozygous allele 1 may elevate serum PGC levels in patients with GC, especially in patients with *H. pylori* infection and intestinal metaplasia [60]. However, Pinto-Correia reported that PGC homozygous allele 1 was related to the up-regulation of PGC expression to serve as a protective factor in the development of gastric disease [61].

Apart from insertion/deletion fragment length polymorphism, the Human Genome Project discovered many *PGC* SNP sites, which also influence PGC expression. He et al. found that healthy subjects who carried the CT and TT genotypes of the rs6912200 polymorphism at the PGC 3′ untranslated region (3′ UTR) had lower histological and serological levels of PGC. The CT or CT/TT genotype of the rs6941539 polymorphism, also located at the PGC 3′ UTR, reduced PGC expression in patients with GC. Besides, two other SNPs, rs9471643 and rs6458238 were reported to play a part in upregulating PGC expression [62–64]. In summary, different gene type of PGC SNP and *H. pylori* infection had effects on susceptibility to GC, which suggested a significant role of PGC SNP in *H. pylori* induced GC. A possible mechanism of polymorphism affecting protein expression is that polymorphic sites in different gene regions may influence promoter activity, binding capability of transcription factors, combination of 3′ UTR and microRNAs (miRNAs) and formation of new splicing variants. Altered PGC expression could at least in part reveal the correlation between individuals with specific genetic variants and GC risk.

#### Epigenetic alteration of PGC gene affects PGC expression

Epigenetic factors, including methylation, acetylation and non-coding RNA, play a crucial role in regulating gene expression. Numerous studies have confirmed that the hypermethylation of a tumour suppressor gene decreases its coding protein expression and subsequent tumorigenesis. Although CpG islands were not present in the *PGC* promoter region under normal conditions, Niwa et al. confirmed that PGC in GC was methylated and that methylation downregulated PGC expression in *N*-methyl-*N*-nitro-*N*-nitrosoguanidine (MNNG)-induced rat GC models. The gastric mucosa of non-cancerous rats and untreated rats were not methylated [65].

Acetylation also affects the expression of PGC. Perlmann et al. found that when an acetylation agent (acetylimidazole) reacts with pepsin, pepsin hydrolysis activity was decreased whereas hydrolysis of the synthetic substrate was increased 2.5-fold. Acetylation of pepsinogen led to a reduced susceptibility of activation, which was reflected through a decreased hydrolysis of the synthetic substrate. Compared to pepsin, the lysine and tyrosine residues of acetylated pepsinogen were acetylated. Therefore, pepsin acetylation was involved in the essential functional groups of enzyme activity, while pepsinogen acetylation mainly affects the conformational characteristics of the molecule [66].

Non-coding miRNAs could bind to the 3′ UTR of their target genes to affect the gene expression. Liu et al. reported that the miRNAs let-7c, let-7i, and let-7f may combine with *PGC* as their target. Serum let-7c is negatively related to *PGC* gene expression [67]. The serum let-7c of individuals with *H. pylori* infection was re-upregulated following *H. pylori* eradication and may induce down-regulation of the PGC expression [68]. Using quantitative reverse transcription-polymerase chain reaction and ELISA assay, Xu et al. found that some serum miRNAs (miR-20a-5p, miR-320a, let-7a) and PGC expression level are positively correlated [69]. These miRNAs may bind to the *PGC* gene 3′ UTR to affect the subsequent PGC expression.

### Hormone regulation of PGC expression

In sex-related tumours such as prostate cancer, breast cancer and ovary cancer, PGC upregulation is an indicator that hormones might play a vital role in regulating PGC expression. Balbin et al. reported that the high PGC expression in breast cancer was the result of hormone changes and was related to the development of breast cancer. Androgen, glucocorticoids and progesterone induced PGC upregulation in breast cancer cells. Incubating breast cancer cells with dihydrotestosterone, progesterone or the synthetic glucocorticoid dexamethasone for 48 h could induce 1.5-kbp *PGC* mRNA aggregation and PGC protein expression. This effect was achieved by the 15-bp cis-acting factor sequence AGAACTATTTGTTCC located in the human *PGC* gene promoter at −444 to −459, which mediates the hormone-induced function of breast cancer cells [51, 70]. Such a hypothesis may be deduced from PGC expression in breast cancer and cysts but not in normal glands. PGC may serve as a biomarker of hormone imbalance under these pathological conditions, reflecting the presence of tumour-associated hormone receptor pathways. Noburo et al. performed immunohistochemical staining after tumour radical prostatectomy and found that only 53% of PGC-negative tumours expressed androgen receptor whereas 87% of PGC-positive tumours expressed androgen receptor [71].

Similarly, in vitro experiments have verified the changes in PGC expression under hormone alteration. Wells et al. found that PGC was expressed in the rat IEC-6 intestinal cell line, and its expression was upregulated under the action of gastrointestinal hormones such as gastrin, secretin and forskolin. The findings demonstrated the role of IEC-6 cells as a model of PGC expression in vitro and in the small intestine [72]. Ishihara screened the rat *PGC* gene using a probe, and found that PGC expression was increased after hydrocortisone injection [73]. Fehrholz induced NCI-H441 airway epithelial cells for 48 h using 1 µM dexamethasone and found that *PGC* mRNA expression levels were increased up to 9.7-fold. Furthermore, adding caffeine for a further 24 h induction resulted in a more notable increase in *PGC* mRNA levels [74]. Dexamethasone promotes the expression of the lung maturation markers SP-B and PGC [17]. It could speculate that glucocorticoids influence human cell maturation and differentiation and increase PGC expression.

The role of hormones such as glucocorticoids, growth factor and hepatocyte growth factor, and mesenchymal interactions are of vital importance for chief cell differentiation. Glucocorticoids influence the gastric epithelial cell proliferation and differentiation in vivo, and might inhibit morphological changes to promote *PG* gene expression. Hydrocortisone treatment of rat gastric explant cells stimulated differentiation, and PG was synthesised. Therefore, glucocorticoid is an important regulatory factor of PGC and of chief cell function and differentiation [75].

### The regulation of PGC expression by other factors

Interactions between PGC with other transcription factors or protein kinases are also involved in the regulation of PGC expression. Sakamoto et al. reported that the 5′ upstream region of the *PGA* and *PGC* genes contains putative transcription factor–binding motifs such as GATA-5 (GATA-binding protein 5), SOX2 (SRY-box 2) and HNF-3β (Hepatocyte Nuclear Factor-3β), which were expressed in intestinal epithelial cells. GATA-5 was highly expressed in chicken gastric mucosa, and efficiently upregulates luciferase gene expression through cis-acting elements. Conversely, SOX2 inhibited PG expression. In addition, chicken PGC expression was associated with epithelial–mesenchymal transition [76, 77]. The transcription factor XBP1 (X-box binding protein 1) regulates the structural alterations of zymogen cells. It could expand the rough endoplasmic reticulum and induce MIST1 (basic helix-loop-helix family member a15) expression to regulate the formation of larger particles in the gastric mucosa. Zymogen cells guided PGC expression in the absence of XBP1 and represented abnormal aggregation of original mucous neck cells [78]. Blandizzi et al. reported that peripheral GABA_B_ (gamma-aminobutyric acid type B receptor unit) receptors mediated an excitatory effect on gastric PG secretion, which depends entirely on acid output [79]. TGFα is best known as a highly potent mitogen, and has been associated with malignant transformation. Sharp and Wang et al. found that MT100 TGFα transgenic mice had a lower level of PGC mRNA expression and a greater decline with the tumor size compared with the nontransgenic mice. It follows that TGFα may directly or indirectly induced decline or depletion of PGC expression [80, 81].

In GC cells, low expressions of mitogen-activated protein kinase activator with WD40 (MAWD, serine/threonine kinase receptor–associated protein) and MAWD-binding protein (MAWBP) were associated with poor survival [82]. Expression of the differentiation-related proteins E-cadherin and PGC was lower in GC tissues than in normal tissues but was upregulated in SGC7901 cells overexpressing MAWD/MAWBP. Wen et al. stimulated MKN74 GC cells using recombinant human bone morphogenetic protein-2 (rhBMP-2) for 48 h, and detected PGC mRNA and protein expression levels. They concluded that rhBMP-2 upregulated PGC expression levels in the cells [83].

## Conclusions and prospects

As a mature sign of organ differentiation, especially gastric differentiation, PGC has unique physiological and pathological characteristics. Currently available researches have suggested that PGC was likely to possess some valuable functions. For example, PGC has antibiotic activity, which might play an antibacterial role in the gastrointestinal mucosa and in gastric mucosa healing. During reproduction, PGC might be helpful for preventing immune infertility, exert local antibacterial action and be involved in the mechanism of preventing infection after sexual intercourse. For all that, we have not yet come to a clear conclusion whether PGC functions as a zymogen. In the future, it is necessary to strengthen research on the special function of zymogenic PGC.

It is known that PGC in situ expression in gastric mucosa is an ideal ‘negative marker’ of GC. Serum PGC and its combination with PGA have satisfactory sensitivity, specificity and price–quality ratio for predicting individuals at high risk of GC. Ectopic PGC expression in prostate cancer, breast cancer, ovary cancer and endometrial cancer indicate better prognosis and longer survival. Regardless of in situ, serum or ectopic expression, PGC demonstrates diagnostic proficiency for distinguishing benign and malignant tumours as well as better or poor prognosis. In addition to serving as a diagnostic marker, PGC might also be a therapeutic target. As a cancer-related marker, monitoring the dynamic changes of PGC expression and cancer occurrence and development is of paramount significance. Currently, the study of PGC’ role in cancer is mainly focused on only the relationship between PGC expression and incidence risk, clinical pathological parameters and prognosis. The applications of PGC in clinical diagnosis and therapy are extremely limited. Translational investigations of the clinical applications of PGC should be enhanced.

As for the regulation of PGC expression, there are a few researches and definite conclusions. Thorough understanding the regulatory factors of PGC expression is particularly advantageous for its better application. In reviewing the studies herein, we found that *PGC* gene polymorphisms regulate PGC expression, and epigenetic alterations affect PGC expression to a certain extent. Ectopic PGC expression is present mainly in sex hormone-related tumours, and we speculate that the absence of PGC expression in GC and sex hormones may have co-effects on GC development or hormone receptor pathways. Whether the absence of PGC expression in GC is associated with changes in hormone levels is currently unknown, therefore there is still a need for in-depth study to clarify the mechanisms of hormone regulation of PGC expression in gastric mucosa. Additionally, although PGC is considered as an effective product of the tumorigenic phase but not a tumour-driven factor, we cannot exclude from the interaction between PGC and other protein factors that PGC might inhibit GC occurrence and development in a negative feedback manner in the complex biological systems of humans. Nevertheless, its potential mechanism still warrants further investigation.

## Abbreviations

PGC: pepsinogen C
PGA: pepsinogen A
PGB: pepsinogen B
PGF: pepsinogen F
SPB: surfactant protein B
hCAP: human cathelicidin
GC: gastric cancer
SG: superficial gastritis
AG: atrophic gastritis
MUC1: mucin 1
*H. pylori*: *Helicobacter pylori*
NCGA: non-cardia gastric adenocarcinoma
IPMN: intraductal papillary mucinous tumours
ESD: esophageal squamous dysplasia
ESCC: esophageal squamous cell carcinoma
CA125: cancer antigen 125
HCC: hepatic carcinoma cells
SNP: single-nucleotide polymorphism
miRNA: microRNA
MNNG: *N*-methyl-*N*-nitro-*N*-nitrosoguanidine
GATA-5: GATA-binding protein 5
SOX2: SRY-box 2
HNF-3β: forkhead box A2
GABAB: gamma-aminobutyric acid type B receptor unit
MAWDBP: MAWD-binding protein
rhBMP-2: recombinant human bone morphogenetic protein-2
